# Editorial: Patho- and Physiological Roles of Inflammasomes

**DOI:** 10.3389/fimmu.2022.857929

**Published:** 2022-02-28

**Authors:** Guangxun Meng, Carsten J. Kirschning, Rongbin Zhou

**Affiliations:** ^1^ Institut Pasteur of Shanghai, Chinese Academy of Sciences (CAS), Shanghai, China; ^2^ Institute of Medical Microbiology, University Clinic Essen, University of Duisburg-Essen, Essen, Germany; ^3^ Department of Life Sciences and Medicine, University of Science and Technology of China, Hefei, China

**Keywords:** inflammasome, innate immunity, pattern recognition receptor, inflammation, physiological inflammation, pathology

Inflammasomes are cytoplasmic multi-protein complexes that mediate pro-cysteine-aspartic protease (caspase)-1 autocleavage, as well as immature interleukin (IL)-1 type cytokine and gasdermin (GSDM) D processing. The consequent pyroptosis results from the integration of pro-GSDMD N-termini into the cell membrane to channel the cellular release of pro-IL-1β and/or -18 C-termini, which are pro-inflammatory cytokines, alongside all inflammasome components. Prototypic inflammasomes encompass a so-called sensor protein such as NLR family pyrin domain containing (NLRP) 3 or are absent in melanoma (AIM) 2, the adaptor protein apoptosis-associated speck-like protein containing a CARD (ASC), and pro-caspase-1. The noncanonical inflammasome employs caspase-4 or -5 in humans and caspase-11 in mice as an LPS-specific pattern recognition receptor (PRR) besides NLRP3, ASC, and caspase-1. The diversity of signals mediating protein complex formation and activation is particularly large in respect to the canonical NLRP3 inflammasome and comprises xenobiotic or host-derived danger indicative molecules. Exemplary inflammasome activation signals are a flux of ions such as K^+^, oxygen radicals, and pathogen or danger associated molecular patterns (P/DAMPs) including LPS, DNA, endocytosed crystals such as uric acid or cholesterol, and ATP. Often as second rather than first line innate immune defenders towards septic or traumatic homeostasis disruption after a first hit-mediated induction of inflammasome-related protein expression, inflammasomes critically contribute to parrying local insult and consequent healing. Systemic challenge driven ubiquitous inflammasome activity or hereditary hyperactivity, however, harm rather than protect an organism. Both inflammasome subfunction and hyperactivity have been implicated as singular or contributory causes of numerous infectious, autoinflammatory, autoimmune, metabolic, and even oncologic disease pathologies.

The collection of articles in this Research Topic on the “*Patho- and Physiological Roles of Inflammasomes*” covers important aspects of inflammasome involvement in various disease conditions while also providing a perspective on its homeostasis protectiveness. Inflammatory bowel disease (IBD) is characterized by chronic and relapsing inflammation in the gastrointestinal tract. Against the background of NLRP3 being a crucial intestinal homeostasis regulator, Zhen and Zhang reviewed the current literature on the function of the NLRP3 inflammasome and its potential roles in the pathogenesis of IBD, and discussed the interaction network connecting NLRP3 with the mucosal immune response and towards upholding gut homeostasis as investigated in experimental animal models and IBD patients’ specimen. Mao et al. reviewed the function of IL-1β, pointing out that both experimental colitis and gut inflammation in humans with IBD are exacerbated when IL-1β secretion is increased. Importantly, for IBD therapy, Crohn’s disease patients lacking CARD8 respond only to treatments involving IL-1β inhibition since in such patients NLRP3 inflammasome activity is increased. Nonetheless, the function of NLRP3 in IBD is still controversial. The differences between results of distinct investigations may result from differences in mouse and human genetic backgrounds, compositions of gut microbiota, as well as colitis models and approaches to induce intestinal inflammation. Context-dependent understanding of the inflammasome’s role in IBD and the potential of modulation of its function awaits further studies.

As a key component of the innate immune system, inflammasomes generally protect the liver against pathogen infection, metabolic syndrome, oxidative stress, and cancer. However, defectively controlled inflammasome activity might promote specific liver pathogenesis. A substantial amount of research has focused on the role of inflammasomes in acute and chronic liver diseases such as viral hepatitis, nanoparticle-induced liver injury, as well as alcoholic and non-alcoholic steatohepatitis. Luan and Ju reviewed the advances of the knowledge on the physiological and pathological roles of inflammasomes in general and in particular NLRP3 in liver diseases and discussed previous reports with respect to the potential development of inflammasome-targeting therapeutics for liver diseases.

Inflammasome dysfunction contributes to sepsis and septic shock approximately 50% of cases of which are caused by Gram-negative bacterial infection involving LPS as major PAMP. The original research article from Chen et al. demonstrates the requirement of advanced glycosylation end-product specific receptor (AGER/RAGE) for caspase-11 inflammasome activation in macrophages. A nuclear P/DAMP complex including high-mobility group box 1 (HMGB1), histone, and DNA promoted caspase-11-mediated GSDMD cleavage, IL-1β proteolysis, and release, as well as cytotoxicity. Inhibition of AGER-mediated lipid peroxidation *via* arachidonate 5-lipoxygenase (ALOX5) limited caspase-11 activation and consequent pyroptosis. This work thus identified novel molecular players in caspase-11 inflammasome activation such as in sepsis.

Recent reports have demonstrated the importance of inflammasome driven and unconventional protein secretion with the presence of alarmins such as galectin-3 and HMGB1 beyond IL-1β and IL-18 cellular release upon inflammasome activation. Cypryk et al. thus focused their review on inflammasome-related unconventional protein secretion while emphasizing vesicle-mediated secretion of a multitude of immunoregulatory proteins, which might represent a novel paradigm in the inflammatory immune response field. This work thus highlighted unsolved questions about metabolic signals and mechanisms by highlighting inflammasome activity including unconventional protein secretion by employing extracellular vesicles. Advanced understanding of the inflammasome-mediated secretion of specific proteins beyond IL-1 type cytokines and inflammasome components may aid therapeutic intervention in inflammatory disease pathologies including those underlying atherosclerosis, arthritis, asthma, and Alzheimer’s disease.

The most studied inflammasome, namely the NLRP3 inflammasome, is involved in the pathogenesis of several diseases. Attribution and identification of pathologic mechanisms elicited by its dysregulation have been controversial. Although quite substantial progress has been achieved during recent years, a unifying model explaining its complex and often paradoxical roles under different disease conditions is still lacking. Inclusion of the aspect of NLRP3 regulation at the transcriptional and post-translational levels, the latter involving protein modification such as by specific kinases and phosphatases being influenced by P/DAMPs including bile acid, might be critical. Therefore, Song and Li reviewed the current literature on NLRP3 phosphorylation patterns and their functional relevance for the regulation of the NLRP3 inflammasome and suggested potential directions for future research.

We express our great gratitude foremost to all authors of this Research Topic, as well as the reviewers for their contributions and insights. We also thank the readers for their interest and support. With effort from many active investigators in the field, breakthroughs in inflammasome research are likely, which could pave the way to preventative or therapeutic interventions in inflammasome-related disorders.

## Author Contributions

GM drafted the initial file. CK edited through with critical input. RZ reviewed the final file. All authors contributed to the article and approved the submitted version.

## Conflict of Interest

The authors declare that the research was conducted in the absence of any commercial or financial relationships that could be construed as a potential conflict of interest.

## Publisher’s Note

All claims expressed in this article are solely those of the authors and do not necessarily represent those of their affiliated organizations, or those of the publisher, the editors and the reviewers. Any product that may be evaluated in this article, or claim that may be made by its manufacturer, is not guaranteed or endorsed by the publisher.

